# TREM1-PET imaging maps whole-body innate immune responses in a mouse model of metastatic melanoma

**DOI:** 10.1038/s41598-026-36542-x

**Published:** 2026-02-26

**Authors:** Irene N. Falk, Aisling M. Chaney, Rohit Verma, Renesmee C. Kuo, Samantha Reyes, Mackenzie Carlson, Mausam Kalita, Carmen Azevedo, Isaac M. Jackson, Jonathan Green, Israt S. Alam, Andrew Tran, Ayush Pant, Emily M. Deal, Michael Lim, Michelle L. James

**Affiliations:** 1 Department of Radiology, Stanford University, Stanford, CA USA; 2 Department of Neurosurgery, Stanford University, Stanford, CA USA; 3 Department of Electrical Engineering, Stanford University, Stanford, USA; 4 Department of Bioengineering, Stanford University, Stanford, CA USA; 5 Department of Neurology and Neurological Sciences, Stanford University, Stanford, CA USA

**Keywords:** Positron-emission tomography (PET), Triggering receptor on myeloid cells-1 (TREM1), Tumor-associated macrophages (TAMs), Tumor-associated myeloid cells (TAMCs), Brain metastases, Tumor microenvironment, Neurology, Oncology, Biomarkers, Predictive markers, Prognostic markers, Cancer imaging, CNS cancer, Metastasis, Tumour biomarkers, Tumour immunology, Imaging the immune system, Immune evasion, Innate immune cells, Neuroimmunology, Tumour immunology

## Abstract

Poor treatment response in brain metastases is largely attributed to anti-tumor T-cell suppression through the modulation of tumor-associated myeloid cells (TAMCs), resulting in immune evasion. Triggering receptor expressed on myeloid cells-1 (TREM1) is a membrane receptor highly expressed on TAMCs that is associated with poor clinical outcomes and of interest as a potential imaging biomarker of myeloid cell function, prognosis, and treatment response. Here we evaluate TREM1-targeted positron emission tomography (PET) tracer, [^64^Cu]TREM1-mAb, for TAMC detection in a murine model of intracranial metastatic melanoma. Forty-eight hours after tracer administration, PET imaging revealed significantly higher [^64^Cu]TREM1-mAb signal in implanted tumors compared to contralateral brain parenchyma or sham brains. *Ex vivo* gamma counting and autoradiography confirmed significantly elevated, tumor-localized signal, while markedly lower uptake with [^64^Cu]-isotype control-mAb confirmed tracer specificity. Similar patterns were seen in the lymphoid organs, including bone marrow and spleen. Flow cytometry confirmed TREM1 expression in myeloid cells alone in brain and spleen. We conclude that [^64^Cu]TREM1-mAb is a promising PET tracer for the detection of increased TREM1^+^ myeloid cells in the tumor microenvironment and peripheral tissues.

**Supplementary Information:**

The online version contains supplementary material available at 10.1038/s41598-026-36542-x.

## Introduction

Metastatic brain tumors represent the most common form of intracranial neoplasm in adults. It is estimated that between 20 and 40% of cancer patients will develop brain metastases each year, equivalent to 100,000–200,000 new diagnoses annually^1–5^. These tumors often present with seizures, paralysis, cognitive decline, and other neurological deficits, leading to significant morbidity and mortality^3,6^. Understanding the mechanisms by which metastatic tumor cells invade brain parenchyma and evade immune surveillance may help to identify predictors of tumor growth and recurrence, and guide strategies for developing and optimizing treatments^7–9^. One mechanism through which metastatic brain tumors have been demonstrated to suppress anti-tumor immune response is via the modulation of tumor-associated myeloid cells (TAMCs) including tumor-associated macrophages (TAMs) and myeloid derived suppressor cells (MDSCs), which subsequently suppress anti-tumor T cell-activation^10^. The ability to monitor TAMCs in the brains and lymphoid tissues of patients with brain metastases may therefore be a valuable prognostic indicator of tumor growth or response to treatment.

Magnetic resonance imaging (MRI) is the current standard of care for the detection and structural characterization of brain tumors^11^, but classical MRI does not directly detect molecular markers that can help identify specific cells and their functional phenotypes in the tumor microenvironment (TME). In contrast, positron emission tomography (PET) is a highly sensitive molecular imaging modality that can be used to noninvasively monitor biochemical and cellular processes *in vivo*^10,12^. Although a wide range of small-molecule PET tracers exist for the detection of neuroinflammatory markers in the brain, most lack true specificity for myeloid cells^12^. ImmunoPET tracers consist of PET radioisotopes conjugated to highly specific antibodies, which provide a means to detect markers of interest with ultra-high specificity and has been proven particularly useful for imaging immune targets^13^. Moreover, emerging artificial intelligence-driven radiomics approaches in PET imaging are being explored to quantitatively extract imaging features that capture tumor heterogeneity and microenvironmental characteristics, offering potential to enhance molecular image interpretation and predictive modeling^14^.

The 18-kDa translocator protein (TSPO) is a widely imaged PET target in neuroinflammation but lacks specificity for a single cell type or lineage. Triggering receptor expressed on myeloid cells 1 (TREM1), in contrast, is a membrane receptor involved in innate immune cell signaling that has been found specifically on TAMCs in a wide range of cancers^15–18^. Notably, TREM1 expression levels have been closely linked to cancer recurrence, metastasis, and poor survival rates^17^, indicating its potential as a biomarker to the functional status of myeloid cells within the TME. A 2022 analysis of transcriptomic signatures in gliomas revealed significantly higher TREM1 expression in high-grade tumors. Similarly, Ma et al. found that increased levels of TREM1 immunostaining of GBM tissue was associated with worse patient prognosis^17^. Moreover, TREM1 is highly expressed on TAMCs but not cancer cells, making it a promising and specific imaging biomarker for detecting TAMCs^15^.

[^64^Cu]TREM1-mAb is highly promising immunoPET tracer that has been previously characterized and validated for its specificity for detecting TREM1 in murine models of multiple sclerosis (MS), ischemic stroke, and Parkinson’s disease (PD)^19–21^. In our previously reported studies, TREM1-PET enabled visualization of innate immune responses in the brain and peripheral tissues. Additionally, we showed that PET signal correlated with disease severity and diminished following immunomodulatory therapy in an MS model. These earlier findings established TREM1-PET as a sensitive and specific imaging approach for monitoring innate immune responses across different neuroinflammatory contexts. Building on this foundation, herein we sought to evaluate the potential of TREM1-PET to noninvasively detect and track tumor-associated TREM1 expression. Specifically, we assessed [^64^Cu]TREM1-mAb for its ability to detect TAMCs in an intracranial model of metastatic melanoma. Our results revealed significantly elevated TREM1-specific PET signal in both the tumor and peripheral lymphoid organs of tumor-bearing mice compared with controls. *Ex vivo* biodistribution studies confirmed increased TREM1-specific tracer accumulation in perfused lymphoid tissues, while autoradiography of tumor-bearing brains revealed higher TREM1-specific signal within the tumor relative to tumor-bearing animals receiving a non-specific isotype control. Flow cytometry further validated these results, showing increased TREM1 expression in TAMCs in both the brain and spleen of tumor-bearing animals. Together, these data establish [^64^Cu]TREM1-mAb as a promising tool for detecting TREM1-mediated immune activity across central and peripheral compartments in brain metastases.

## Results

### TREM1-PET signal is significantly elevated in brain tumors compared to normal brain tissue

**Fig. 1:**
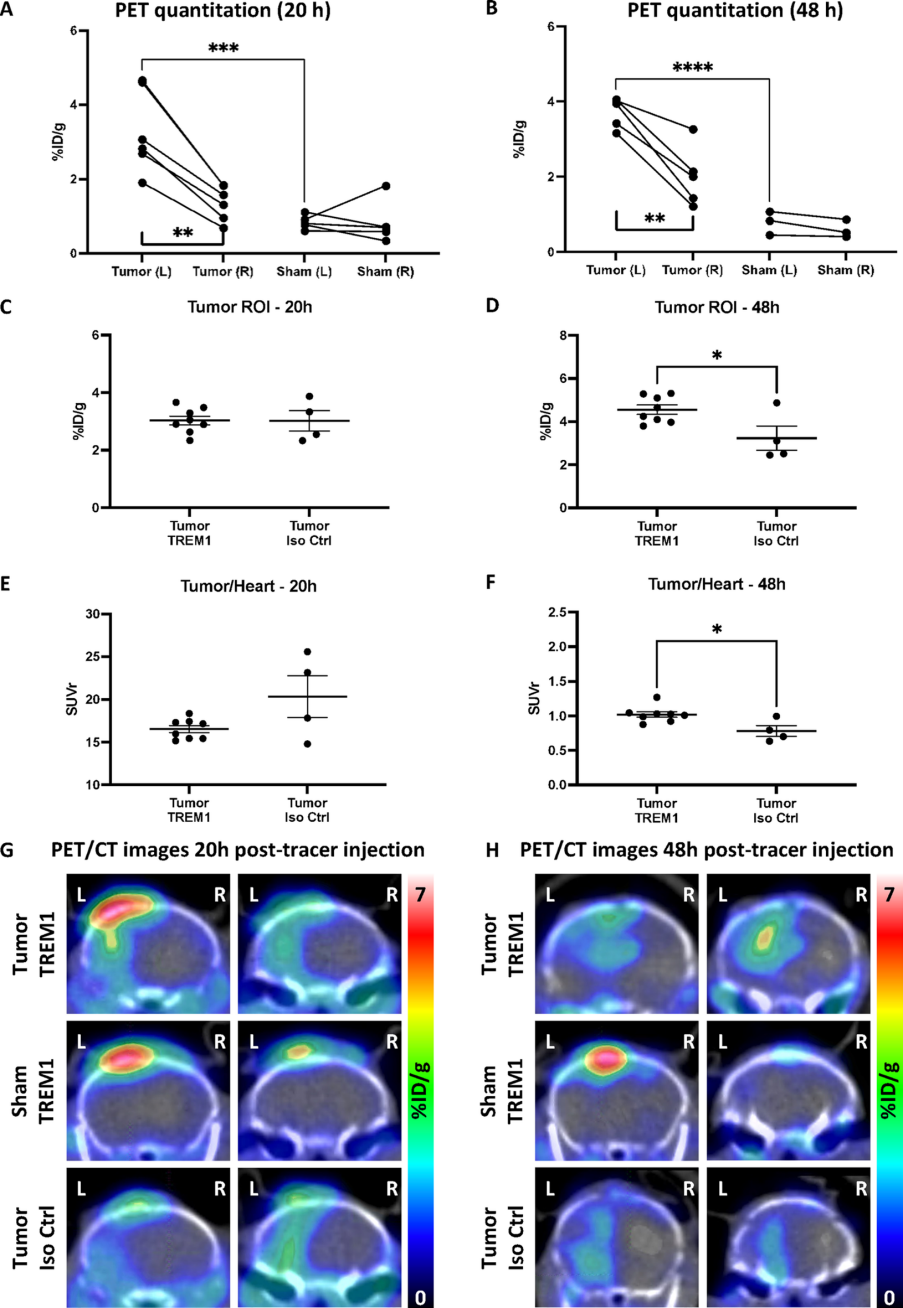
Quantitation of PET signal (%ID/g) in left tumor or sham-injected hemisphere relative to the contralateral right hemisphere at (**A**) 20 h and (**B**) 48 h post-tracer injection in tumor-bearing mice vs. sham mice. Regions of interest defining the tumor were established using T2-weighted-MRI as an anatomical guide after co-registration with PET/CT. (**C–D**) Comparison of [^64^Cu]TREM1-mAb (“TREM1”) vs. [^64^Cu]-isotype control-mAb (“Iso Ctrl”) PET signal from tumor ROI in tumor-bearing animals that received [^64^Cu]TREM1-mAb vs. [^64^Cu]-isotype control-mAb at (**C**) 20 h and (**D**) 48 h post-tracer injection. (**E–F**) Comparison of tumor/heart ROI signal ratios in tumor-bearing animals that received [^64^Cu]TREM1-mAb vs. [^64^Cu]-isotype control-mAb at (**E**) 20 h and (**F**) 48 h post-injection. (**G–H**) Representative coronal PET-CT images at (**G**) 20 h and (**H**) 48 h post-tracer injection in a tumor-bearing mouse injected with [^64^Cu]TREM1-mAb vs. a sham mouse injected with [^64^Cu]TREM1-mAb vs. a [^64^Cu]-isotype control-mAb-injected tumor-bearing mouse. Error bars represent the standard error of the mean. *: *p* < 0.05; **: *p* < 0.01; ***: *p* < 0.001; unpaired t tests.

We first assessed whether [^64^Cu]TREM1-mAb could effectively detect elevated TAMCs in the brains of tumor-bearing mice by comparing PET signal intensities between hemispheres vs. signal intensities in sham-operated mice. PET/CT imaging was performed at 20 and 48 h after tracer injection. At 20 h post-tracer injection, TREM1-PET signal was significantly higher in the left hemisphere of tumor-bearing mice compared to contralateral parenchyma (*n* = 6) and compared to the corresponding left brain hemisphere of sham mice (*n* = 5) (tumor hemisphere: 3.72 ± 0.18% of injected dose per gram [%ID/g]; contralateral: 2.01 ± 0.35%ID/g; sham: 0.78 ± 0.18%ID/g, *p* < 0.001, Fig. 1A). Similar differences were observed at 48 h, with a significant elevation in signal in the ipsilateral hemisphere vs. the contralateral hemisphere of tumor-bearing mice (*p* < 0.0001, Fig. 1B).

Next, we quantified TREM1-PET signal localized specifically within the tumor region, using T2-weighted (T2w) MRI and histology to define the tumor region of interest (ROI; Figure S2). To assess the specificity of the TREM1-PET tracer in this model, we compared the tumor ROI signal in mice injected with [^64^Cu]TREM1-mAb vs. mice administered a radiolabeled isotype control tracer. Notably, tracer signal within the tumor ROI was significantly higher in tumor-bearing mice than in a corresponding ROI in sham controls, consistent with whole-hemisphere analysis. At 20 h post-injection, there was no significant difference in tumor ROI PET signal between tumor-bearing-mice imaged with [^64^Cu]TREM1-mAb (*n* = 15) vs. [^64^Cu]-isotype control-mAb (*n* = 5) (*p* = 0.98; Fig. 1C). However, at 48 h post-injection, tumor ROI PET signal was significantly higher in tumor-bearing mice that received [^64^Cu]TREM1-mAb (*p* = 0.023, Fig. 1D).

To better correct for signal originating from circulating mAb PET tracer, we calculated standardized uptake value ratios (SUVr) by normalizing the tumor PET signal to PET signal from the blood pool using an ROI drawn over the heart. At 20 h post-injection, no significant difference was observed in tumor/heart signal ratios between tumor-bearing mice imaged with [^64^Cu]TREM1-mAb vs. [^64^Cu]-isotype control-mAb (*p* = 0.055; Fig. 1E). However, at 48 h post-injection, tumor/heart SUVr was significantly elevated in mice that received [^64^Cu]TREM1-mAb, indicating higher TREM1-specific signal within the tumors at this later timepoint (*p* = 0.013, Fig. 1F).

Representative coronal PET/CT images acquired at 20 (Fig. 1G) and 48 h (Fig. 1H) post-injection reflect these findings, highlighting the markedly increased tracer binding at the tumor site in mice injected with [^64^Cu]TREM1-mAb compared with both sham controls and tumor-bearing mice receiving the isotype control tracer. Notably, in sham and tumor-bearing mice injected with [^64^Cu]TREM1-mAb, elevated signal was also observed near the skull surface at the incision site. This localized uptake was more pronounced than in tumor-bearing mice that received the isotype control, suggesting that the TREM1-PET tracer specifically detects inflammation associated with surgical injury, consistent with its known binding to activated myeloid populations.

### TREM1-PET signal is increased in lymphoid tissues of animals with intracranial tumors

**Fig. 2:**
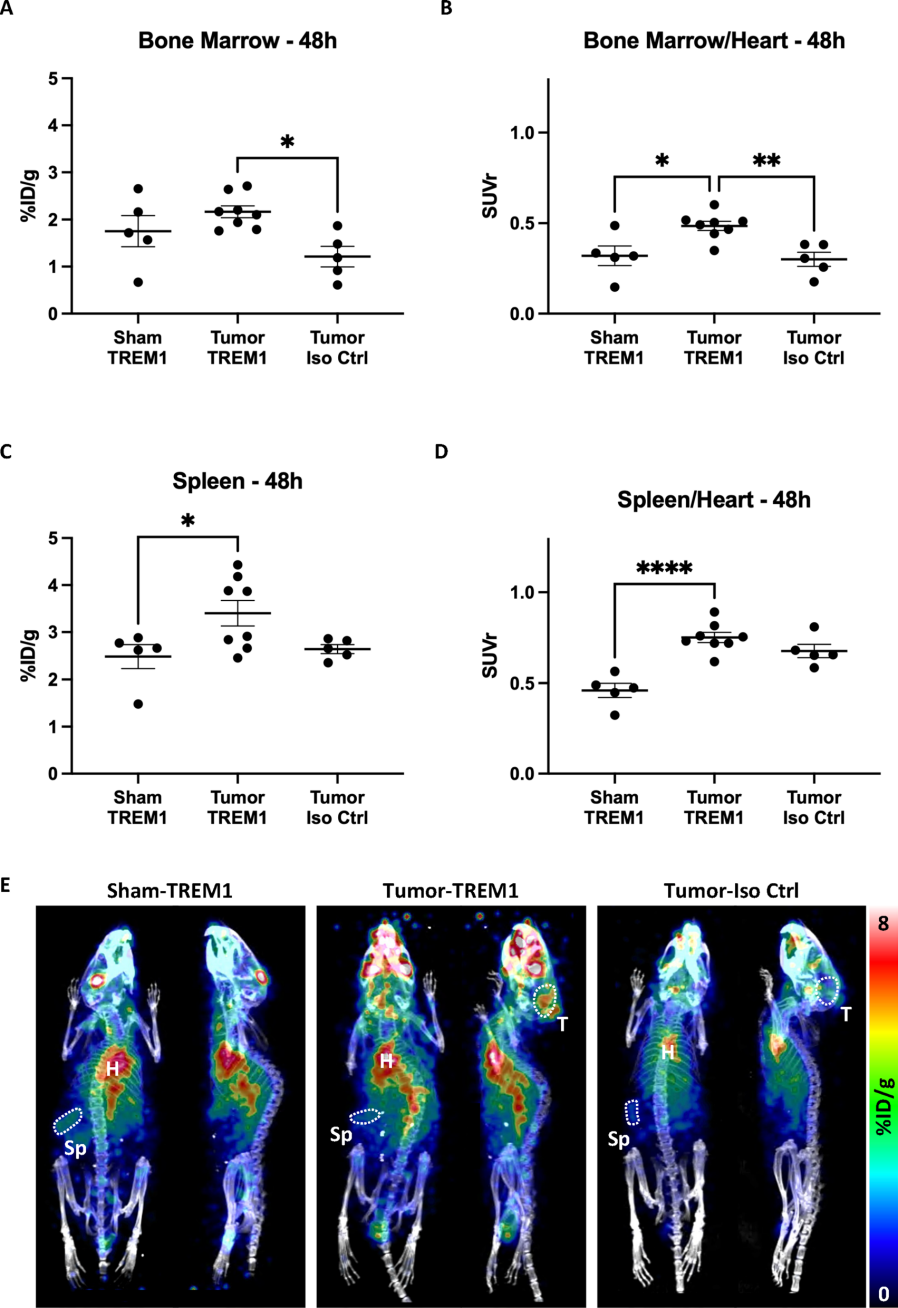
(**A**) PET signal (%ID/g) and (**B**) PET SUVr from the bone marrow of sham mice injected with [^64^Cu]TREM1-mAb, intracranial tumor-bearing mice injected with [^64^Cu]TREM1-mAb, and intracranial tumor mice injected with [^64^Cu]-isotype control-mAb. (**C**) PET signal (%ID/g) and (**D**) PET SUVr from the spleens of sham mice injected with [^64^Cu]TREM1-mAb, intracranial tumor-bearing mice injected with [^64^Cu]TREM1-mAb, and intracranial tumor-bearing mice injected with [^64^Cu]-isotype control-mAb. (**E**) Representative whole-body images from the aforementioned groups. *: Error bars represent the standard error of the mean. *: *p* < 0.05; **: *p* < 0.01; ****: *p* < 0.0001; one-way ANOVA with Tukey’s multiple comparisons tests.

To quantify TREM1-specific PET signal in key lymphoid tissues typically involved in tumor-immune responses^22^, we analyzed PET signal in the spleen and bone marrow using a combination of manually drawn ROIs and previously reported thresholding techniques^23^. In tumor-bearing animals that received [^64^Cu]TREM1-mAb, %ID/g values were elevated in bone marrow compared to tumor-bearing animals receiving the isotype control (*p* = 0.011), but not significantly different from sham animals injected with [^64^Cu]TREM1-mAb (*p* = 0.32; Fig. 2A). To more accurately measure regional differences in TREM1 expression level, we applied an image-derived input function correction, normalizing tissue PET signals to the blood pool to generate standardized uptake value ratios (SUVr). Bone marrow SUVr in tumor-bearing mice that received [^64^Cu]TREM1-mAb were significantly higher than those in both sham controls and tumor-bearing animals injected with [^64^Cu]-isotype control-mAb (*p* = 0.012 and *p* = 0.0058, respectively; Fig. 2B). While group differences in spleen %ID/g PET signal were modest (*p* = 0.035 vs. sham controls and *p* = 0.083 vs. isotype controls; Fig. 2C), spleen SUVr were significantly elevated in tumor-bearing animals receiving [^64^Cu]TREM1-mAb relative to sham animals (*p* < 0.0001, Fig. 2D). Representative whole-body PET images for each mouse group at 24 and 48 h post tracer injection demonstrated elevated bone marrow signal in the femurs of tumor-bearing mice injected with [^64^Cu]TREM1-mAb, without grossly visible differences in spleen signal across groups (Fig. 2E and S3), aligning with our quantitative analysis.

### Gamma counting demonstrates significantly higher TREM1-specific tracer binding in lymphoid tissues of animals with intracranial tumors

**Fig. 3:**
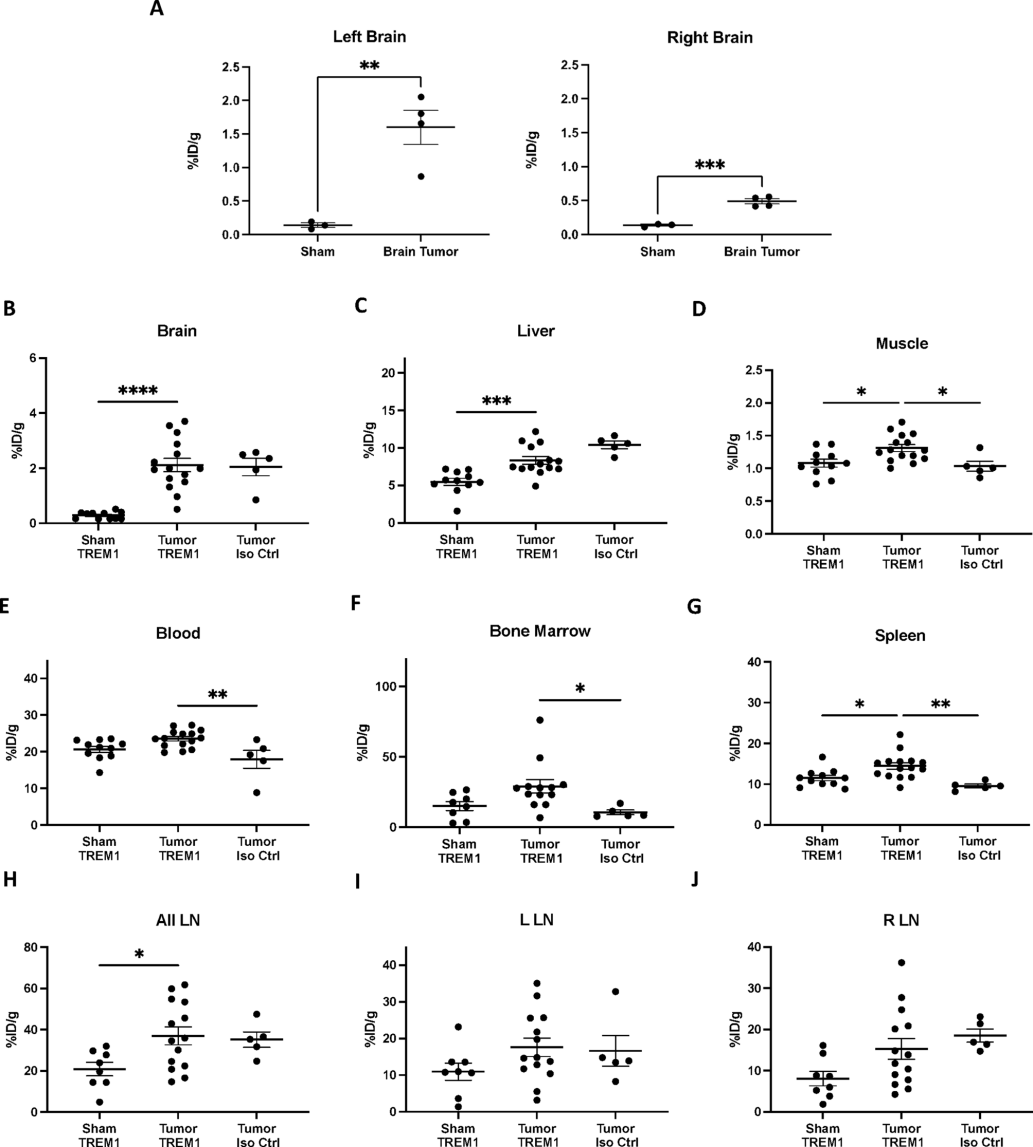
(**A**) Biodistribution analysis of the tumor-bearing left hemispheres and contralateral right hemispheres of tumor-bearing animals compared to the corresponding hemispheres of sham mice. Gamma counting results (%ID/g) from the (**B**) brains, (**C**) liver, (**D**) quadriceps femoris muscle, (**E**) blood, (**F**) bone marrow, (**G**) spleens, and (**H**–**I**) right and left cervical lymph nodes of sham mice injected with [^64^Cu]TREM1-mAb, intracranial tumor-bearing mice injected with [^64^Cu]TREM1-mAb, and intracranial tumor-bearing mice injected with [^64^Cu]-isotype control-mAb. Error bars represent the standard error of the mean. *: *p* < 0.05; **: *p* < 0.01; ***: *p* < 0.001; ****: *p* < 0.0001; (**A**) Mann–Whitney tests; (**B**–**J**) one-way ANOVA with Tukey’s multiple comparisons tests.

To account for the known shortcomings of small animal PET imaging (e.g., limited spatial resolution and partial volume effects) which can impact the accuracy of quantifying tracer binding in narrow organs or small regions (e.g., in spleen or lymph nodes), we dissected each tissue of interest for *ex vivo* gamma counting and compared the resulting signal to our *in vivo* imaging results. Gamma counting analysis revealed higher levels of tracer in the left and right brain hemispheres of tumor-implanted mice compared to sham mice (left: *p* < 0.01, right: *p* < 0.001, Fig. 3A). Additionally, *whole*-brain gamma counting demonstrated significantly higher PET signal in tumor-bearing mice relative to sham mice that received [^64^Cu]TREM1-mAb (*p* < 0.0001). However, there was no significant difference in [^64^Cu]TREM1-mAb tracer signal in whole brain of tumor-bearing mice compared to those injected with [^64^Cu]-isotype control-mAb (Fig. 3B).

Peripheral biodistribution studies revealed significant elevations in TREM1 tracer signal across multiple tissues in tumor-bearing mice compared with sham and isotype control mice. For example, liver signal was increased in tumor-bearing mice injected with the TREM1 tracer relative to sham mice, and tumor-bearing mice receiving the isotype control also showed higher liver uptake, this difference was not statistically significance (Fig. 3C). TREM1-specific signal was also significantly elevated in the blood, bone marrow, muscle, and spleen of tumor-bearing animals that received [^64^Cu]TREM1-mAb compared with tumor-bearing mice injected with [^64^Cu]-isotype control-mAb (Fig. 3D**–**G). In contrast, although tracer binding differed significantly between sham and tumor-bearing mice, no significant asymmetry was observed between the right and left cervical lymph nodes in either group (Fig. 3H**–**J).

### Autoradiography confirms increased specific binding of [^64^Cu]TREM1-mAb in the TME

**Fig. 4:**
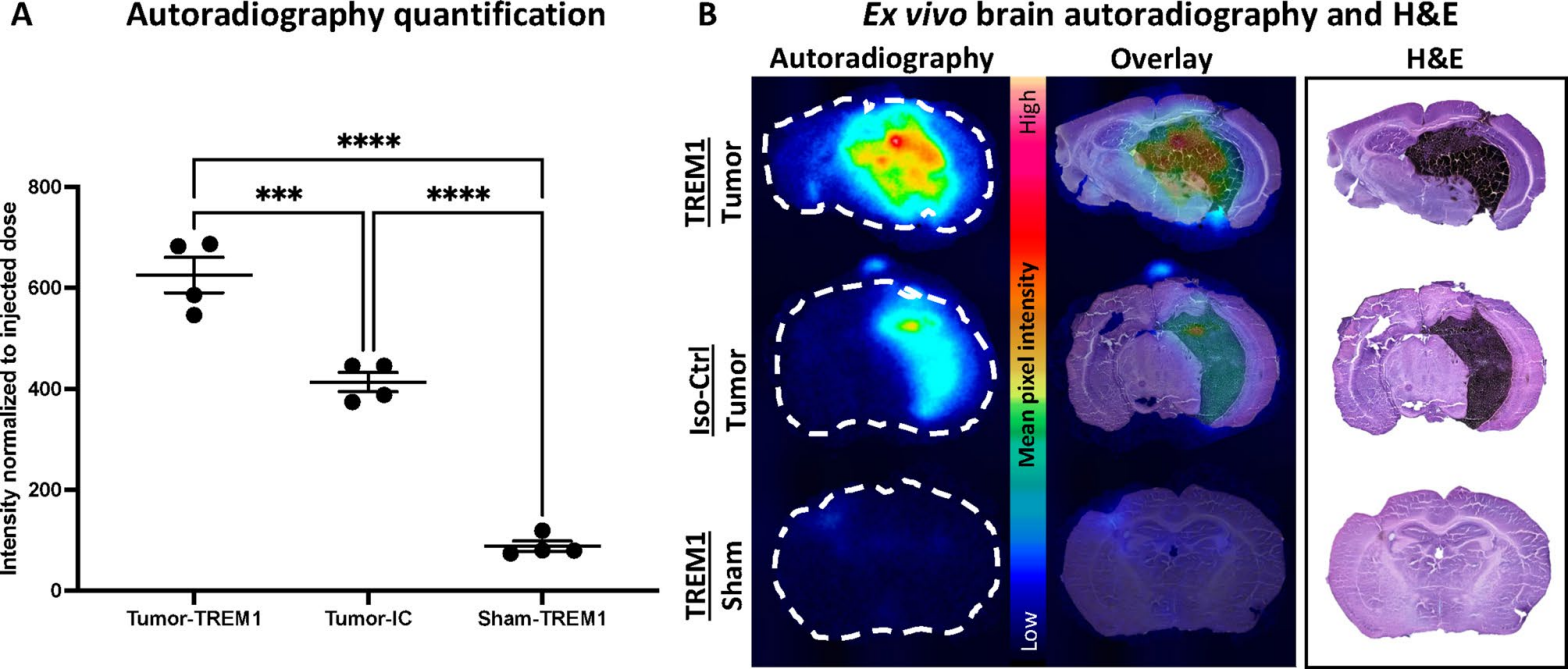
Quantitative autoradiography of **(A)** the tumor ROI from tumor-bearing mice vs. whole sections of control brain. **(B)** High-resolution *ex vivo* autoradiography and H&E staining of 40 μm-thick coronal brain sections from tumor-bearing and sham mice injected with [^64^Cu]TREM1-mAb and tumor-bearing mice injected with [^64^Cu]-isotype control-mAb. White-dashed lines outline the coronal brain sections. Error bars represent the standard error of the mean. ***: *p* < 0.001; ****: *p* < 0.0001; one-way ANOVA with Tukey’s multiple comparisons tests.

*Ex vivo* autoradiography (ARG) of fresh-frozen brain tissue sections obtained 48 h post-injection revealed significantly greater [^64^Cu]TREM1-mAb binding in tumor-bearing mice compared with both sham and isotype control groups (*p* < 0.001; Fig. 4A). Binding of the [^64^Cu]-isotype control-mAb in tumor-bearing brains was markedly lower than that of [^64^Cu]TREM1-mAb (*p* < 0.001), confirming the specificity of the TREM1 tracer in the TME. Notably, elevated [^64^Cu]TREM1-mAb signal localized precisely to the tumor region, as confirmed by H&E staining of the same tissue sections (Fig. 4B).

### Increased TREM1 expression in tumor-bearing mice localizes to an expanded population of CD45^hi^CD11b^+^ myeloid cells in the brain and spleen

**Fig. 5:**
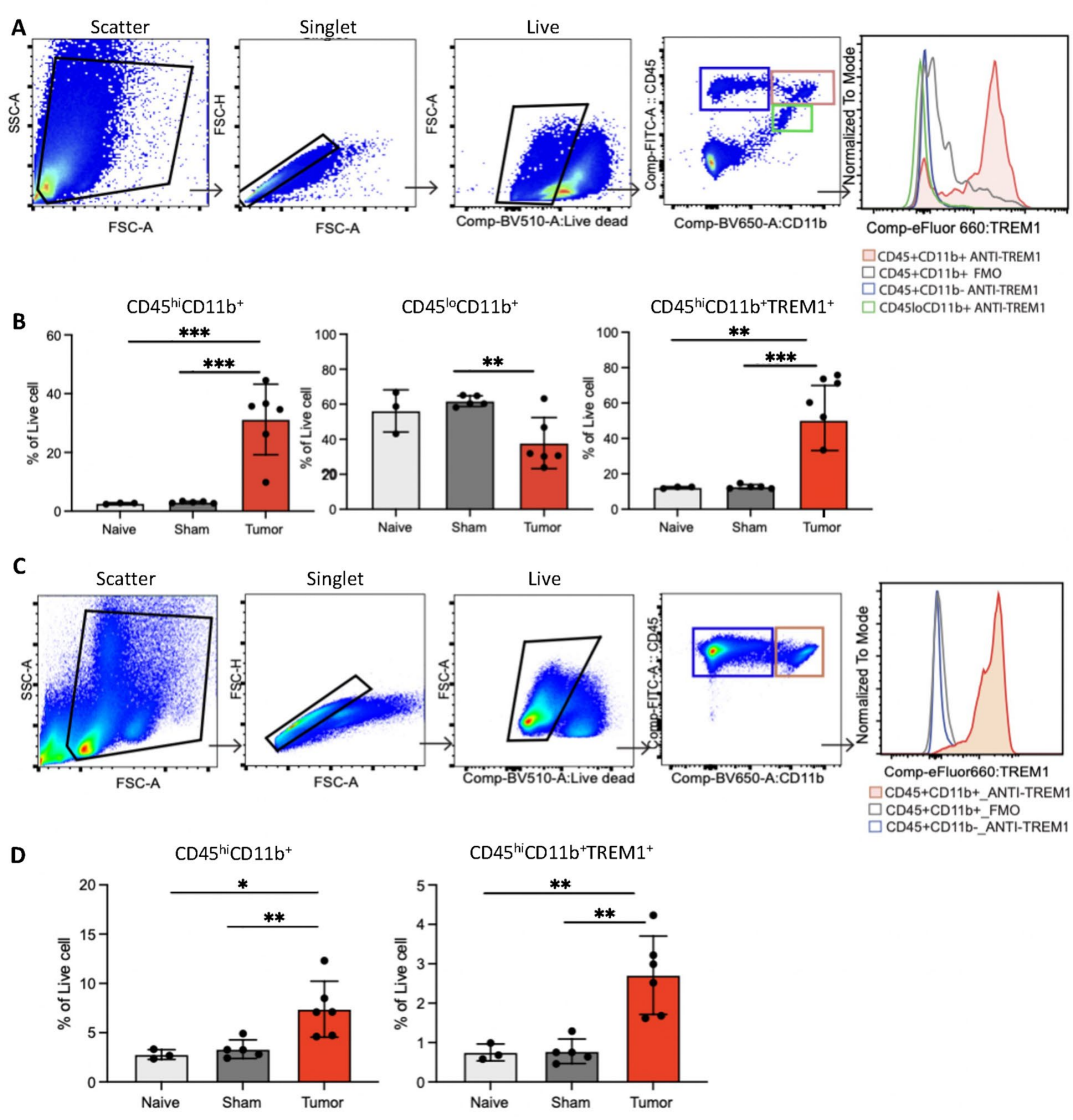
(**A**) Gating strategy for analysis of immune populations from the brains of tumor mice. (**B**) Bar graphs showing percentage of CD45^+^CD11b^+^ and CD45^lo^ CD11b^+^ and TREM1^+^ immune cells in the brains of naïve, sham and tumor-bearing mice. (**C**) Gating strategy for analysis of immune populations from the spleens of tumor mice. (**D**) Bar graphs showing percentage of CD45^+^CD11b^+^ and CD45^lo^ CD11b^+^ and TREM1^+^ immune cells present in brains of naïve, sham and tumor-bearing mice. Error bars represent the standard error of the mean. **: *p* < 0.01; **: *p* < 0.001; one-way ANOVA with Tukey’s multiple comparisons tests.

Flow cytometry analyses of immune cells from the brains and spleens of tumor-bearing animals revealed significant increases in TREM1^+^CD45^hi^CD11b^+^ myeloid cell populations. High surface expression of TREM1 was found in CD45^hi^CD11b^+^ myeloid cells, but virtually no expression was found in CD45^lo^CD11b^+^ microglia or CD45^hi^CD11b^−^ lymphocytes (Fig. 5A-B). Similarly, TREM1 expression in the spleen was identified in CD45^hi^CD11b^+^ myeloid cells but not CD45^hi^CD11b^−^ cells (Fig. 5C-D). Further analysis of Ly6c and Ly6g expression for the identification of granulocytic vs. monocytic myeloid-derived suppressor cells (MDSCs) revealed higher rates of TREM1 expression among granulocytic MDSCs (Figure S4).

### TREM1 is expressed on myeloid cells in human brain metastasis

**Fig. 6:**
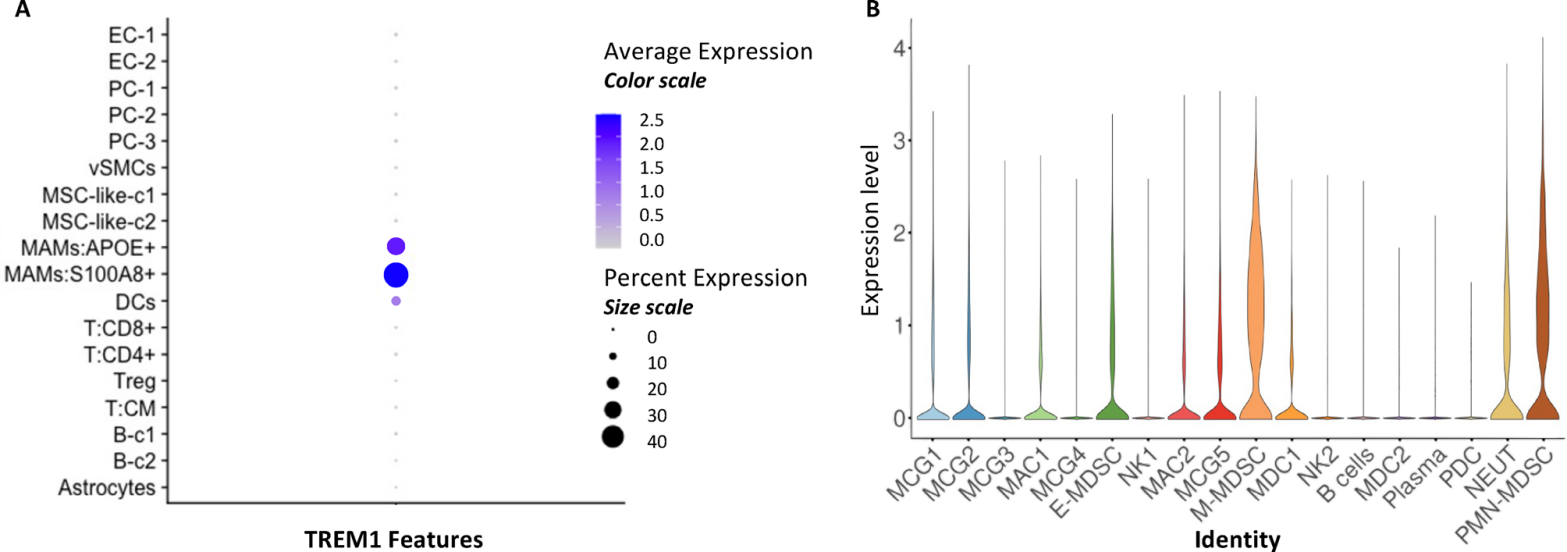
(**A**) Dot plot of percentage and average expression of TREM1 RNA in different cell types present in human brain metastasis. **(B**) Violin plot showing expression of TREM1 at the single cell level.

Finally, we investigated the expression of Trem1 in human brain metastases in a publicly available single cell dataset. Single cell RNA (sRNA) sequencing data contained in this database revealed higher expression of Trem1 in s100A8^+^ and APOE-expressing human myeloid cells in the context of brain metastases, whereas no expression was detected in cancer cells or lymphocyte immune subsets, including both T and B cells in these human brain tissue samples (Fig. 6).

## Discussion

It is well established that a robust TAMC population forms a critical part of the immunosuppressive TME in both human brain tumors and murine intracranial cancer models^24^. TREM1 is a signaling receptor highly and specifically upregulated on TAMCs^15^, and its expression has been strongly associated with high-grade malignancy and poor clinical prognosis in patients with brain metastases^17^. Building on this foundation, we investigated the ability of a TREM1-targeted PET tracer to noninvasively detect TAMC activity within the TME of a murine intracranial melanoma model. Using PET imaging, gamma counting, autoradiography, and flow cytometry, we demonstrate that TREM1-PET enables visualization of TAMC-associated immune activity *in vivo*. Notably, elevated tracer binding in both the tumor and peripheral lymphoid organs highlights the potential of TREM1-PET to capture not only localized but also systemic immune responses to cancer.

Our initial PET and *ex vivo* biodistribution studies found significantly elevated PET signal and gamma counts in the brains of tumor-bearing animals when compared to sham animals. While PET signal from tumor ROI was not significantly different between tumor-bearing-mice imaged with [^64^Cu]TREM1-mAb vs. [^64^Cu]-isotype control-mAb at 20 h post-injection, a significant elevation was detected in mice receiving the TREM1 tracer at 48 h post-injection, after additional time for clearance of unbound tracer circulating in the blood. Comparison of autoradiographic images and H&E-stained sections from 40 μm brain slices showed concordance between autoradiography and H&E staining, with areas of high radioactivity corresponding spatially to areas of tumor infiltration. This data was further supported by flow cytometry, confirming myeloid-specific TREM1 expression and an increased frequency of TREM1^+^ myeloid cells in the brains of tumor-bearing mice when compared to shams.

Notably, we also identified increased TREM1-PET signal in lymphoid tissues of tumor-bearing vs. sham animals, suggesting an enhanced peripheral immune response in this model, TREM1-PET SUVr in bone marrow showed significant TREM1-specific signal elevations in tumor-bearing animals when normalized to the blood pool signal, and *ex vivo* gamma counting confirmed elevated TREM1-specific tracer binding. TREM1-PET SUVr were also significantly higher in spleen ROIs from tumor-bearing animals that received [^64^Cu]TREM1-mAb compared with sham controls. While isotype control signal was modestly elevated in the spleens of tumor animals, *ex vivo* gamma counting–a more accurate means to assess tracer binding in murine spleen–ultimately confirmed a TREM1-specific increase. Moreover, flow cytometry analysis corroborated these findings, showing significantly increased TREM1 expression localized to myeloid cells, including MDSCs, in the spleens of tumor-bearing animals relative to sham mice.

This pattern suggests that the elevated TREM1-PET signal in bone marrow and spleen reflects a tumor-associated immune response, whereby signaling from the TME induces systemic TREM1 activation in peripheral myeloid compartments. Similar brain-periphery immune communication has been demonstrated in our previous TREM1-PET studies in murine models of MS, ischemic stroke, and PD^19–21^. In the current intracranial melanoma model, we observed a modest increase in TREM1-PET signal within the spleen and bone marrow, in addition to robust signal in the brain tumor, suggesting a systemic immune component secondary to tumor-driven inflammation. The absence of corresponding signal in sham mice further confirms that these peripheral changes are tumor-driven. In contrast, in our studies of EAE mice (MS model), where disease was induced peripherally via subcutaneous MOG_35 − 55_ immunization, we observed prominent TREM1-PET signal in both the bone marrow and spleen, as well as in the spinal cord, reflecting widespread activation of peripheral myeloid cells preceding central nervous system infiltration. Moreover, in a large-vessel ischemic stroke model, we found elevated tracer signal in the infarcted brain region, spleen, and intestine, but not in bone marrow or blood, indicating a distinct axis of neuroimmune signaling. Meanwhile, in the PD model characterized by dopaminergic neurodegeneration, we found marked TREM1-PET signal in the brain, spleen, and blood, consistent with chronic low-grade systemic inflammation accompanying progressive neuronal loss. Collectively, these findings demonstrate that TREM1-PET sensitively captures both central and peripheral immune activation across diverse pathophysiologic contexts. In the setting of brain metastases, this capability may enable noninvasive monitoring of the dynamic interplay between tumor-associated inflammation in the brain and systemic myeloid activation, providing a more integrated view of whole-body immune responses in cancer.

*Ex vivo* gamma counting also confirmed TREM1-specific signal in other peripheral tissues from tumor-bearing animals, including the blood and muscle, suggesting a widespread innate immune response following intracranial implantation of melanoma cells. Increased specific TREM1 tracer signal in the blood likely reflects higher levels of circulating TREM1-positive myeloid cells in tumor-bearing animals^25^, as well as a component of elevated soluble TREM1^26^. These findings are consistent with published studies describing an increase in MDSCs in blood, bone marrow, and spleen in multiple rodent tumor models as well as in the blood of patients with different cancers^27^. Elevated gamma counts in the liver of tumor-bearing mice imaged with [^64^Cu]TREM1-mAb relative to sham mice also likely reflect increased TREM1-specific tracer binding to myeloid cells. The even higher signal in the liver of tumor-bearing mice injected with isotype control PET tracer is likely due to elevated metabolism, as this antibody has no true specific binding and is thus more available to be broken down and excreted when compared to the TREM1-specific tracer.

While the *in vivo* detection of TREM1-specific signal in this mouse model was sometimes challenging due to variability in specific and non-specific tracer signal, this variability was most likely attributable to the spatial resolution limitations of small-animal PET, the presence of unbound circulating antibodies in hyperemic or highly vascular tissues, and varying degrees of tracer binding to circulating myeloid cells or free tracer in blood. For example, increased signal was observed in the scalp overlying the tumor implantation site, spillover of which could potentially affect accurate quantification of tumor ROI signals in mice injected with the isotype control tracer, particularly at earlier timepoints. However, this signal was cleared between 20- and 48-hour imaging, consistent with a decrease in circulating antibody rather than specific or non-specific tissue binding. While local scalp hyperemia might be observed in patients with prior neurosurgical procedures, this spillover is less relevant in human imaging due to larger anatomical scale. Similarly, while TREM1-PET SUVr values were not significantly elevated relative to isotype control in the spleen, the thin geometry of the mouse spleen introduces partial volume effects that complicate accurate signal quantification^28,29^. This effect is also less pronounced in human clinical imaging, where organ size is larger.

As tumor implantation can disrupt blood-brain barrier (BBB) integrity and lead to enhanced tracer permeability and non-specific retention, we investigated the potential contribution of this effect by including tumor-bearing mice injected with an isotype control antibody lacking TREM1-binding capacity. The marked difference in tumor signal between the TREM1-targeted and isotype control tracers indicate that the observed TREM1-PET signal primarily reflects specific tracer binding rather than passive leakage due to BBB compromise. Moreover, the negligible PET signal observed in the spleen and bone marrow of isotype control-injected tumor-bearing mice further supports the specificity of the TREM1 tracer. TREM1-specific signal was also confirmed by *ex vivo* gamma counting after tissue perfusion to remove the contribution of tracer signal in blood, which is a more accurate means of quantifying tracer concentration in each tissue. The finding of TREM1-specific signal elevations in tumor ROI-PET analysis and high-spatial resolution autoradiography, but not in whole-brain gamma counting, further suggests a dilutional effect in volumetric averaging. The high level of non-specific tracer signal in whole brains of tumor-bearing mice that received the isotype control likely reflects antibody retention in more highly vascular structures, such as the cerebellum, rendering it more difficult to detect TREM1-specific signal elevations associated with the tumor. Future studies with repetitive blood sampling may be useful to better characterize circulating antibody clearance kinetics and improve quantitative modeling of tracer dynamics.

It is possible that increased PET signal in animals that received the isotype control is the result of BBB compromise secondary to malignancy, particularly given increased Fc receptor expression in the TME. While the lack of persistently elevated signal on ARG after perfusion and dissection makes Fc-mediated binding a less likely source of non-specific signal in our model, further optimization of the tracer could improve *in vivo* specificity for TREM1 detection in the TME. One potential strategy involving substituting the full-length TREM1 monoclonal antibody with smaller antibody fragments, such as monovalent F(ab) or divalent F(ab’)_2_ constructs that lack the Fc domain responsible for Fcγ receptor engagement, thereby reducing non-specific binding and background signal^30^. Alternatively, single-chain variable fragments (scFv)^31^ or nanobodies, which retain only the antigen-binding regions^32^, could further enhance tissue penetration and minimize Fc-related interactions. Such approaches may be especially valuable in tumors where Fc receptor expression is upregulated^33,34^, and could ultimately advance the use of TREM1-PET for therapy monitoring in malignancies.

Cumulatively, our PET imaging, gamma counting, autoradiography and flow cytometry results demonstrate that our tracer enables detection of elevated TREM1^+^ cell populations within both the TME and peripheral lymphoid tissues of tumor-bearing mice. These results establish the potential of TREM1-PET imaging with [^64^Cu]TREM1-mAb as a sensitive tool for visualizing and monitoring maladaptive immune response in both intracranial and systemic compartments. Unlike the widely used TSPO imaging biomarker, which is expressed across multiple cell types including microglia, macrophages, endothelial, and cancer cells, TREM1 provides greater specificity for myeloid-lineage populations. Although our current tracer targets murine TREM1, our findings underscore the translational promise of TREM1 as a clinically relevant biomarker capable of illuminating the dynamic roles of TAMCs and peripheral myeloid cells in brain metastasis progression. In the clinical setting, TREM1-PET could in the future serve as a noninvasive endpoint for evaluating immunotherapeutic efficacy, offering earlier and more precise indicators of treatment response and ultimately guiding strategies to improve outcomes for patients with brain metastases.

## Methods

### Experimental overview

Tracer binding was assessed by PET/CT imaging of mice with orthotopically implanted intracranial melanoma at 20 and 48 h after tracer injection, which correspond to days 7 and 8 after tumor versus sham inoculation (Figure S1). Specificity of the TREM1 tracer for TAMCs was verified by comparison with an isotype control tracer. Tumor PET signal was identified by co-registering brain PET and T2-weighted MR images. After imaging, mice were perfused to remove unbound intravascular [^64^Cu]TREM1-mAb, and radioactivity in dissected tissues was measured using gamma counting. Brain sections from selected tumor and sham mice were further analyzed via *ex vivo* autoradiography and stained with hematoxylin & eosin (H&E) to better visualize tumor shape and size. Flow cytometry was used to confirm elevated TREM1 expression on myeloid cells in the TME of this mouse model.

### Animal care

All animal care and procedures were performed in compliance with the Animal Welfare Act, in accordance with institutional guidelines, and with approval by the Stanford Administrative Panel on Laboratory Animal Care, which is accredited by the Association for the Assessment and Accreditation of Laboratory Animal Care International. Study design is illustrated in Figure S1. Female 6–8-week-old C57BL/6J wildtype mice (Jackson Laboratory strain 000664) were housed in a temperature-controlled environment under a 12-hour light/dark schedule with unrestricted access to food and water. For intracranial procedures, animals were anesthetized via intraperitoneal injection of ketamine (80 mg/kg) and xylazine (8 mg/kg). For imaging studies, animals were anesthetized with inhaled isoflurane (1–3%) and maintained on isoflurane at 1–2% for the duration of the experiment. Animal numbers are described in Table 1.

**Table 1:** Summary of animal numbers per group. Some animals died or required euthanasia between the 20-hour and 48-hour PET scans. Organ tissues that were not properly perfused or showed evidence of hemorrhagic injury were excluded.

TREM1-brain tumor	TREM1-Sham	Iso Ctrl- brain tumor
15	12	5

### Cell culture

Murine B16-F10 expressing with luciferase (B16-luc) was procured from ATCC and cultured in Dulbecco’s Modified Eagle Medium (DMEM, Gibco) with 10% fetal bovine serum (FBS, Sigma-Aldrich) and 1% penicillin-streptomycin (Sigma-Aldrich). All cell lines were kept in a 37 °C humidified incubator with 5% CO_2_. For tumor implantation, cells were trypsinized using 0.05% trypsin-EDTA (Gibco) and washed in phosphate buffered saline (PBS, Gibco). Viability and quantity were assessed using an automated cell counter and 0.4% Trypan Blue (Gibco) staining. Cells were resuspended at a final concentration of 25,000 cells/1µL PBS for implantation.

### Tumor implantation

For intracranial tumor implantation, mice were anesthetized via intraperitoneal injection with ketamine-xylazine solution. The surgical area was sanitized, and surface of the skull exposed via a small midline incision. A left-sided burr hole was drilled at the following coordinates: 2 mm anterior and 2 mm lateral to lambda. B16-luc cells (50,000) in 2µL DMEM were stereotactically implanted into the left striatum, 3 mm deep to the cortical surface. Detailed methodology has been previously described^35^. Seven days post-implantation, luciferin was injected intraperitoneally, and tumor presence was confirmed using an In Vitro Imaging System (PerkinElmer).

### Bioluminescent imaging (BLI)

On day 5 post-tumor inoculation, sham- and tumor-inoculated mice received intraperitoneal injections of luciferin (150 µL, 15 mg/mL in PBS) and were imaged on an IVIS Spectrum (Caliper Life Science). Signal was imaged using Living Image 4.0 software.

1,4,7,10-Tetraazacyclododecane-1,4,7,10-tetraacetic acid (DOTA) conjugation

### 1,4,7,10-Tetraazacyclododecane-1,4,7,10-tetraacetic acid (DOTA) conjugation

Anti-rat TREM1-mAb and isotype-control-mAb (Rat IgG2A Clone #174031, R&D) were conjugated with DOTA according to standard procedures using metal-free buffers. In brief, a solution of DOTA-NHS ester (Macrocyclics Inc.) in dimethyl sulfoxide (25 mmol/L; 9–12 µL) was added to 1 ml of HEPES buffer (0.1 mol/L, pH 8.8) containing 500 µg of TREM1-mAb or isotype-control-mAb, and the reaction mixture was incubated at 4 °C overnight. The reaction was quenched with Tris pH 7.4 (Sigma), excess DOTA-NHS was removed by Zeba Spin Desalting Columns (0.5 ml, 70 K molecular weight cut-off, ThermoFisher Scientific) and the resulting solution was buffer-exchanged into ammonium acetate buffer (0.1 M, pH 5.5) for ^64^Cu labeling. DOTA-conjugate solutions were concentrated by ultrafiltration (Vivaspin 2 mL, Sartorius) to 1–3 mg/mL, snap-frozen in liquid nitrogen and stored at − 80 °C before radiolabeling. The number of DOTA chelators coupled per antibody was estimated to be between 2 and 4 for both TREM1 and isotype-control, measured via matrix-assisted laser desorption/ionization-time of flight MS, by comparison with unconjugated mAb versus DOTA-conjugated mAb.

### Radiometal-labeling

Both DOTA-TREM1-mAb and DOTA-isotype-control-mAb were radiolabeled with ^64^Cu (*t*_½_ = 12.7 h) using previously described methods with some modifications. DOTA-TREM1-mAb/DOTA-isotype-control-mAb (100 µg) in 30–50 µl of 0.25 mol/L ammonium acetate buffer (0.1 M, pH 5.5) was mixed with pH-balanced ^64^CuCl_2_ solution (44–74 MBq, pH 4.5-5.0, University of Wisconsin or Washington University in St. Louis) at 37 °C with gentle shaking at 400 rpm. Radiolabeling was monitored via thin layer chromatography (TLC) and upon completion of the reaction (30–60 min), 0.1 M EDTA (0.5 M, pH 8.0) was added to a final concentration of 0.01 M and incubated for 15 min to scavenge unchelated ^64^CuCl_2_ in the reaction mixture. Purification of each radiolabeled antibody was achieved by G25 Sephadex size-exclusion purification (NAP-5 column). Radiochemical purity was determined by instant TLC with TEC-Control Chromatography strips (Biodex Medical Systems), developed in saline, and size-exclusion liquid chromatography with a Phenomenex Section 3000 column (Torrance) with sodium phosophate buffer (0.1 mol/L, pH 6.8) at a flow rate of 1.0 mL/min. ^64^Cu-labeled anti-TREM1-mAb (that is, [^64^Cu]TREM1-mAb) and [^64^Cu]-labeled isotype-control-mAb (that is, [^64^Cu]-isotype control-mAb) were obtained with high specific radioactivity (> 0.400 MBq/µg), radiochemical purity (> 99%) and labeling efficiency (70–95%) and formulated in phosphate-buffered saline (0.1 mol/L NaCl, 0.05 mol/L sodium phosphate [pH 7.4]).

### Radiotracer injection and image acquisition

[^64^Cu]TREM1-mAb (230 ± 7 µCi) or [^64^Cu]isotype control-mAb (226 ± 1.3 µCi) was administered via tail vein injection to anaesthetized tumor-bearing animals on day 6 post-tumor inoculation. Similar dosages of [^64^Cu]TREM1-mAb (214 ± 14 µCi) were administered to sham animals at day 6 post-sham surgery.

PET/CT and MR imaging was acquired at 20 and 48 h after tracer injection. Static PET data was acquired in list mode format throughout the 10 min scan using the GNEXT scanner (Sofie), which delivers 0.54 mm isotropic spatial resolution at the center of a 130 mm field of view. Isotropic resolution was achieved using OSEM3D reconstruction algorithms with 24 subsets, 3 iterations, and a matrix size of 240 × 240 × 191. CT scans were collected on the GNEXT to provide attenuation correction and an anatomic reference for the PET data.

PET/CT images were analyzed using VivoQuant 4.0 (InVicro). PET was coregistered with CT images for anatomical reference. Brain regions were analyzed using VivoQuant’s semi-automatic brain atlas tool and percent injected dose per gram (%ID/g) was calculated from the mean signal in each brain region, normalized to the total decay-corrected dose to each mouse.

Bone marrow regions of interest (ROIs) were isolated by Otsu thresholding following previously published methods^23,36^, where the outline of left femur was used to delineate the position on the bone marrow.

Following PET/CT scanning, animals underwent head-and-neck MRI using an actively-shielded Bruker 7T horizontal bore scanner (Bruker Corp, Billerica MA), with International Electric Co. (IECO) gradient drivers, a 120 mm ID shielded gradient insert (600 mT/m, 1000 T/m/s), AVANCE III electronics; 8-channel multi-coil RF and multinuclear capabilities and volume RF coils; and the supporting Paravision 6.0.1 platform. The facility provides isoflurane anesthesia in medical grade oxygen, and physiological monitoring of the subject including electrocardiogram (ECG), pulse oximetry, respiration, and temperature feedback for core body temperature maintenance by warm airflow over the animal. T2-coronal and axial images were acquired with the following parameters: echo time: 33 ms; repetition time: 2,500 ms; 2 averages; 17 slices with thickness 0.5 mm and voxel size of 0.25 mm x 0.1556 mm x 0.5 mm.

### Biodistribution

Following PET/CT/MR imaging on day 8, cardiac puncture was performed under anesthesia with inhaled isoflurane (2–3%). Mice were subsequently perfused with 20–40 mL PBS to remove unbound intravascular tracer. The following tissues were dissected and immediately weighed for gamma counting to measure radioactivity per gram: blood, bone marrow, brain, cervical lymph nodes, heart, liver, muscle (quadriceps femoris), and spleens. The tail was also removed and analyzed for radioactive content to correct for the amount of the tracer dose that was successfully delivered intravenously versus that which remained in the tail. Results were computed as %ID/g, using the weight of each dissected organ.

### Autoradiography

Following imaging, [^64^Cu]TREM1-mAb binding distribution in the CNS was evaluated using high-resolution *ex vivo* autoradiography (ARG). Dissected brains and brain hemispheres were immediately transferred into Optimal Cutting Temperature (OCT) compound (Tissue Tek) and frozen. Specimens were subsequently cut into 40 μm coronal sections in a cryostat (Thermo Scientific Microm HM550) at a temperature of -14 to -16 °C and slide-mounted. Slides were then exposed to a digital film (BAS-IP SR 2025; Fujifilm) for 120–130 h (equivalent to at least 10 half-lives for the ^64^Cu isotope). Following exposure, ROIs were manually drawn and mean pixel intensity was normalized to background mean pixel intensity.

The same section used for ARG were stained with hematoxylin and eosin (H&E; Hematoxylin Gills 3, Thermo Scientific #72604; Eosin-Y, Richard-Allan Scientific, Thermo Scientific #71204) for anatomical reference, from which ROIs were manually drawn.

### Flow cytometry

On day 8, brains and spleen were harvested in complete RPMI and mechanically dissociated with a 3 ml syringe plunger on 70 μm strainer (Corning) to produce a single-cell suspension. Brain single cell suspensions were centrifuged through a continuous 30% Percoll gradient to remove myelin and other cell debris. Pellets were resuspended in FACS buffer and stained with Ghost live-dead Fixable Cell Stain for 5 min (Tongo) to exclude dead cells. Cells were then stained for CD45 (BioLegend), CD3 (BD Bioscience), CD4 (Thermo Fischer), CD8 (BioLegend), CD11b (BioLegend), CD11c (BioLegend), F4/80 (BioLegend), B220 (Biolegend) Ly6C (BioLegend), Ly6G (BioLegend), Trem1 (Thermofisher). Samples were acquired using a CYTEK aurora (CYTEK Bioscience). Data was analyzed using FlowJo (BD).

Single cell RNA seq analysis of publicly available data for TREM1 expression

### Single cell RNA seq analysis of publicly available data for TREM1 expression

Single-cell RNA-sequencing data from Gonzalez. et al.1 GSE186344 was reanalyzed for this study. Clustering resolution and cell annotation methods were previously described in Gonzalez. et al. Counts were normalized to the total UMI count by cell and log scaled using Seurat,2. These normalized counts were used for TREM1 expression analysis in each cluster.

### Statistics

All statistical tests were conducted using GraphPad Prism (v9.01). For tests involving only two groups, unpaired t tests or Mann-Whitney tests were used. For tests involving three groups, one-way ANOVA with Tukey’s multiple comparisons tests were used.

### Ethics statement

All experiments were conducted in accordance with ARRIVE guidelines. Animal numbers were determined based on prior studies investigating this radiotracer^19^, and mice were randomized into tumor vs. sham groups. Following bioluminescent tumor imaging, tumor-bearing mice were divided into two groups with a similar range of tumor sizes and randomized to receive either the [^64^Cu]TREM1-mAb (“TREM1”) or [^64^Cu]-isotype control-mAb control. Data analysis was blinded where possible (e.g., when comparing tumor-bearing groups). All mice were housed at the James H. Clark Center facilities in accordance with the standards of the American Association for Accreditation of Laboratory Animal Care and the Stanford’s Administrative Panel on Laboratory Animal Care (APLAC). All experiments adhered to a protocol that was reviewed and approved by the Stanford APLAC.

## Supplementary Information

Below is the link to the electronic supplementary material.

Supplementary Material 1

## Data Availability

Data is provided within the manuscript or supplementary information files. Raw data were generated at Stanford University. Derived data supporting the findings of this study are available from the corresponding author MLJ on request.
