# Supplementary material for: TREM1-PET imaging maps whole-body innate immune responses in a mouse model of metastatic melanoma
Source: Sci Rep. 2026 Feb 26;16:11157. doi: 10.1038/s41598-026-36542-x (PMC13046965; doi:10.1038/s41598-026-36542-x)
Supplement: Supplementary file 1 — Supplementary Material 1 [file 41598_2026_36542_MOESM1_ESM.docx]

**Supplemental Materials**

**TREM1-PET imaging maps whole-body innate immune responses in a mouse model of metastatic melanoma**

Irene N. Falk^1^, Aisling M. Chaney^1^, Rohit Verma^2^, Renesmee C. Kuo^1,3^, Samantha Reyes^1^, Mackenzie Carlson^4^, Mausam Kalita^1^, Carmen Azevedo^1^, Isaac M. Jackson^1^, Jonathan Green^1^, Israt S. Alam^1^, Andrew Tran^2^, Ayush Pant^2^, Emily M. Deal^1^, Michael Lim^2^, Michelle L. James^1,5*^

^1^Department of Radiology, Stanford University, Stanford, CA; ^2^Department of Neurosurgery, Stanford University, Stanford, CA; ^3^Department of Electrical Engineering, Stanford University, Stanford; ^4^Department of Bioengineering, Stanford University, Stanford, CA; ^5^Department of Neurology & Neurological Sciences, Stanford University, Stanford, CA.

**Supplementary Figure 1.** Experimental procedure. Stereotactic implantation of tumor cells vs. PBS occurred on day 0, followed by bioluminescent imaging to detect tumor cells on day 5, injection of radiotracer on day 6, PET/CT and MR imaging at 20 and 48 hours after radiotracer injection (days 7 and 8, respectively), followed by animal sacrifice after cardiac puncture and perfusion on day 8 for *ex vivo* gamma counting, autoradiography, and histological analysis of select organs.

**Supplementary Figure 2.** Tumor volume measurements in mice injected with either [^64^Cu]TREM1-mAb or [^64^Cu]-isotype control-mAb. *: Error bars represent the standard error of the mean. ^ns^: p> 0.05; unpaired t-tests were used to evaluate statistical significance.

**Supplementary Figure 3.** Representative whole-body PET images acquired 20 hours after tracer injection of sham mice, tumor-bearing mice injected with [^64^Cu]TREM1-mAb, and tumor-bearing mice injected with [^64^Cu]-isotype control-mAb.

**Supplemental Figure 4.** Representative plots of CD45^+^CD11b^+^TREM1^+^ cells in the brain **A**) and spleen **B**) of tumor-bearing mice analyzed for expression of monocyte and myeloid-derived suppressor cell markers Ly6C and Ly6G, respectively.
